# A Morel-Lavallee Lesion After Total Knee Arthroplasty and Aggressive Physical Therapy Manipulation: A Case Report and Review of the Literature

**DOI:** 10.7759/cureus.96115

**Published:** 2025-11-04

**Authors:** Ioannis G Spyrou, Panayiotis K Karampinas, Evangelos Sakellariou, Periklis Peladis, Aggelos Kontos, Anestis Mitkas, Spyros G Pneumaticos

**Affiliations:** 1 3rd Department of Orthopaedic Surgery, National and Kapodistrian University of Athens, KAT General Hospital, Athens, GRC

**Keywords:** closed degloving, complication of treatment, morel-lavallée, quilting suture, total joint arthroplasties

## Abstract

Total knee arthroplasty (TKA) is one of the most common procedures performed in orthopedics. It has the potential for multiple complications. We present a case of a patient who developed a Morel-Lavallée lesion (closed degloving injury) of the knee in the setting of a postoperative seroma after aggressive physical therapy manipulation following TKA. Clinically, the presentation was similar to a prosthetic joint infection. The patient was treated with thorough open irrigation, debridement, and dead space management with internal quilting sutures, followed by the placement of negative pressure wound therapy and compression, all of which are mainstays of treatment for closed degloving injuries. Despite the rarity of the lesion, the surgeon should maintain a high level of suspicion, even in the setting of seemingly minor repetitive trauma.

## Introduction

Total knee arthroplasty (TKA) is one of the most common procedures performed in orthopedics. In 2008, 615,050 TKAs were performed in adult patients in the United States, and in 2013, it was estimated that about four million people were living with a knee replacement in the United States [1,2]. Despite the effectiveness of the procedure, there are multiple potential complications. For wound-related complications in particular, data suggest surgical site infection rates requiring oral or intravenous antibiotics of up to 4.2% and 0.2%, respectively [3]. Postoperative swelling is more frequent, occurring in 15.6% of patients [3]. Hematomas and seromas have the potential for bacterial contamination, endangering the prosthetic joint [4]. A closed degloving injury, also known as a Morel-Lavallée lesion (MLL), is the separation of the fascia from the overlying subcutaneous tissue when tangential forces are applied. It is a rare entity [5] and is more commonly encountered in traumatic settings where shearing forces are at play. Hence, it has not been recognized as a cause of postoperative knee pain and swelling.

We report the case of a patient who underwent a primary TKA at another institution and, after rigorous knee manipulation and muscle stretching, presented at our clinic 12 weeks later with an MLL of the knee. This was treated with open debridement and closure of the potential dead space with internal quilting sutures. Although postoperative swelling and effusion are common after TKA, no case of a closed degloving injury in this context has been reported in the literature.

## Case presentation

**Figure 1:**
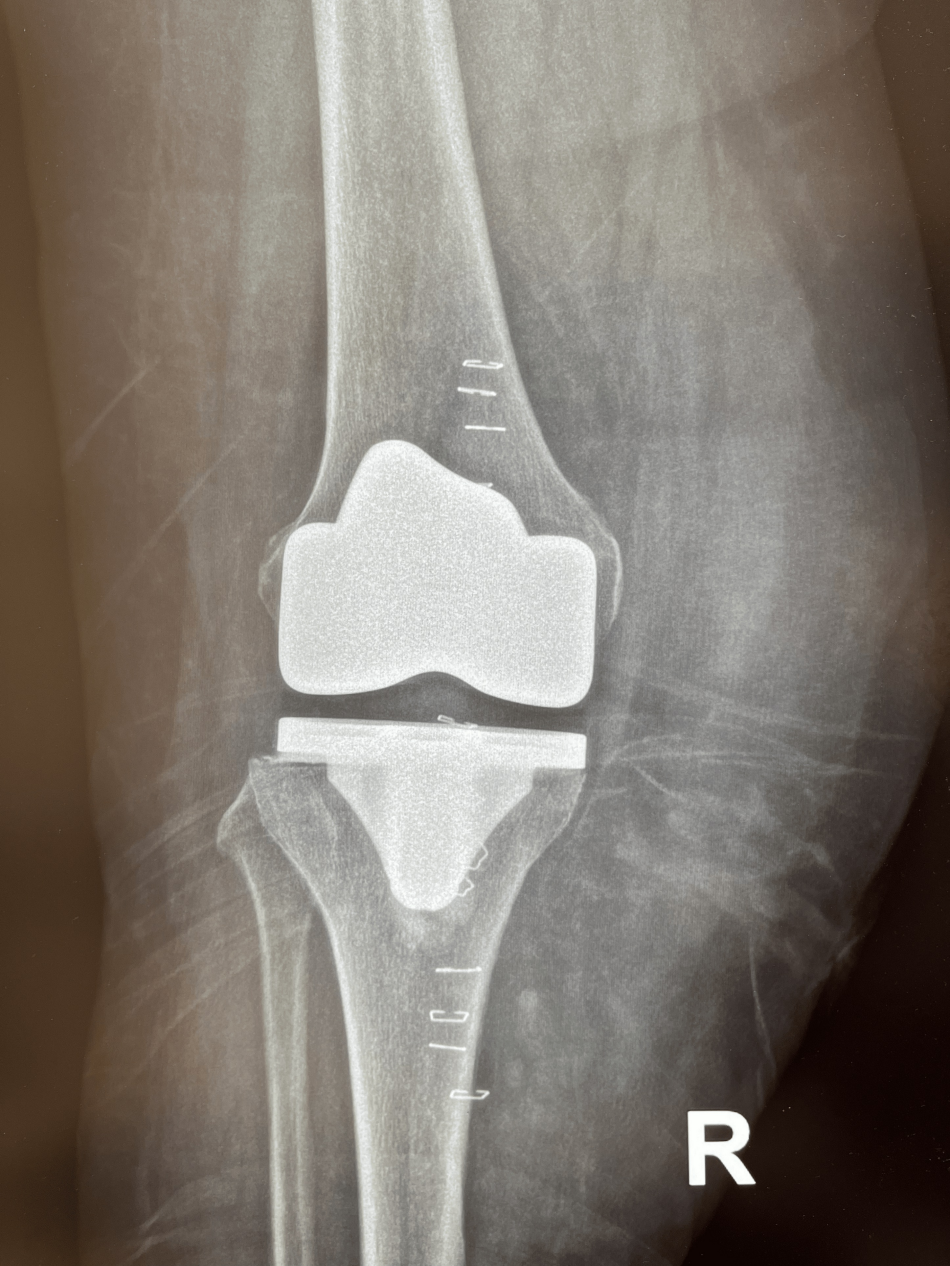
AP (anteroposterior) radiograph of the knee at presentation

**Figure 2:**
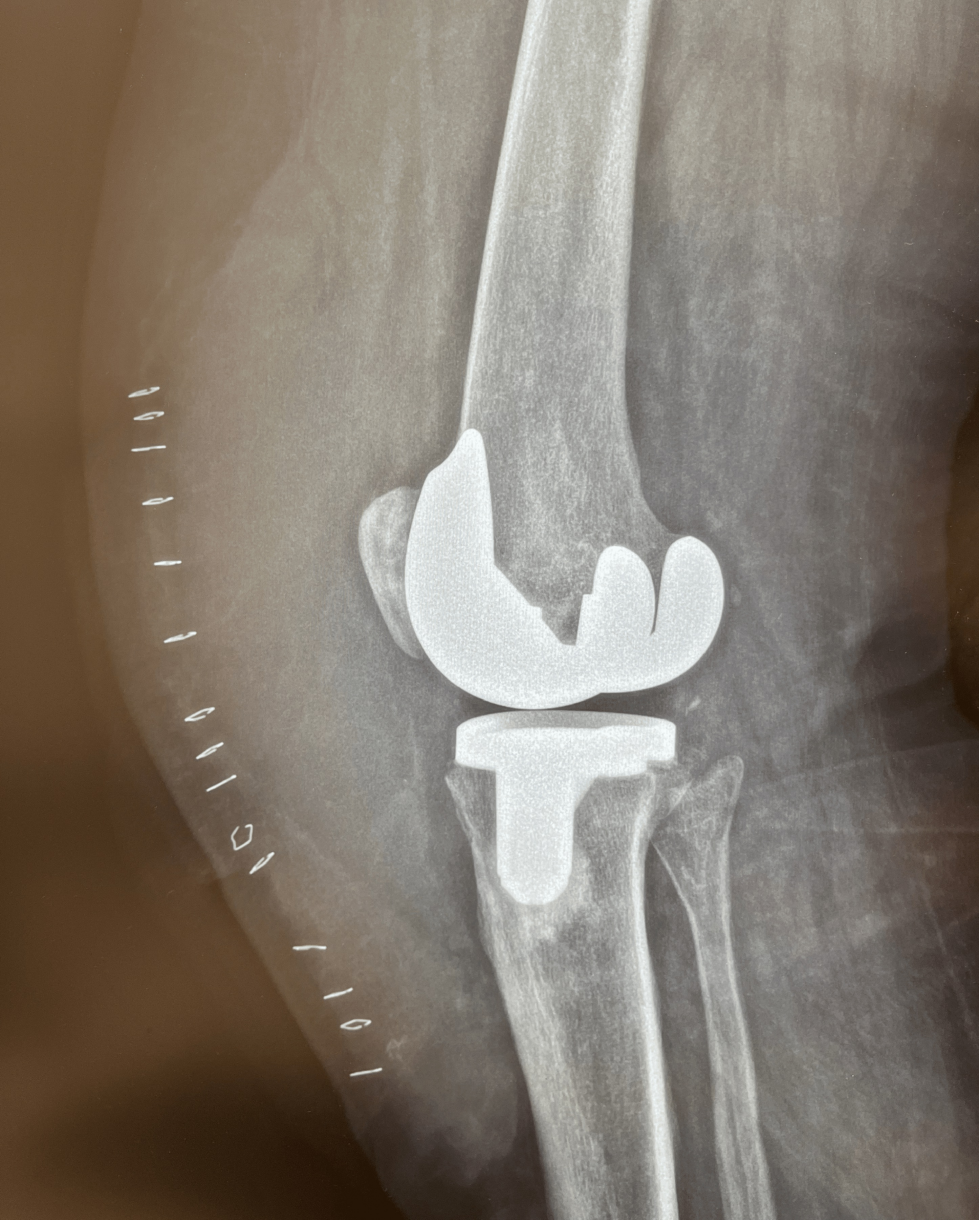
Lateral radiograph of the knee at presentation

A 67-year-old female patient presented to our outpatient clinic 12 weeks after undergoing a primary TKA of the right knee at another institution. The patient had undergone open irrigation and debridement (I&D) 10 weeks postoperatively at the latter, including polyethylene exchange and tissue sampling for cultures. Her past medical history was remarkable for diabetes mellitus, arterial hypertension, and atrial fibrillation (on oral anticoagulants). Macroscopically, the joint showed significant swelling, slight warmth, and a painful range of motion, raising concern for a prosthetic joint infection. The patient was admitted to our department (Orthopedic Department, Level I trauma center) for further investigation and treatment. C-reactive protein (CRP) was negative (<0.31 mg/dL). The patient reported that her postoperative course at the original institution was uneventful. However, even after four weeks of physical therapy, there was joint stiffness and restricted range of motion. After the I&D, the patient had received a short course of oral antibiotics. Furthermore, the patient recalled that, due to mild joint swelling and stiffness, the physical therapist had performed rigorous joint manipulation with an instrument resembling a rolling pin for massaging the muscles around the joint. Radiographs showed marked soft-tissue swelling (Figures 1, 2). The patient reported that intraoperative cultures from the previous surgery were negative. The knee was aspirated, and the fluid was sent for culture.

While the inflammatory markers were not elevated and the patient was afebrile, another matter of concern was that significant time had passed since the index procedure; therefore, the decision was made to address the problem operatively, and surgery was scheduled seven days later. The knee aspiration culture was also negative.

**Figure 3:**
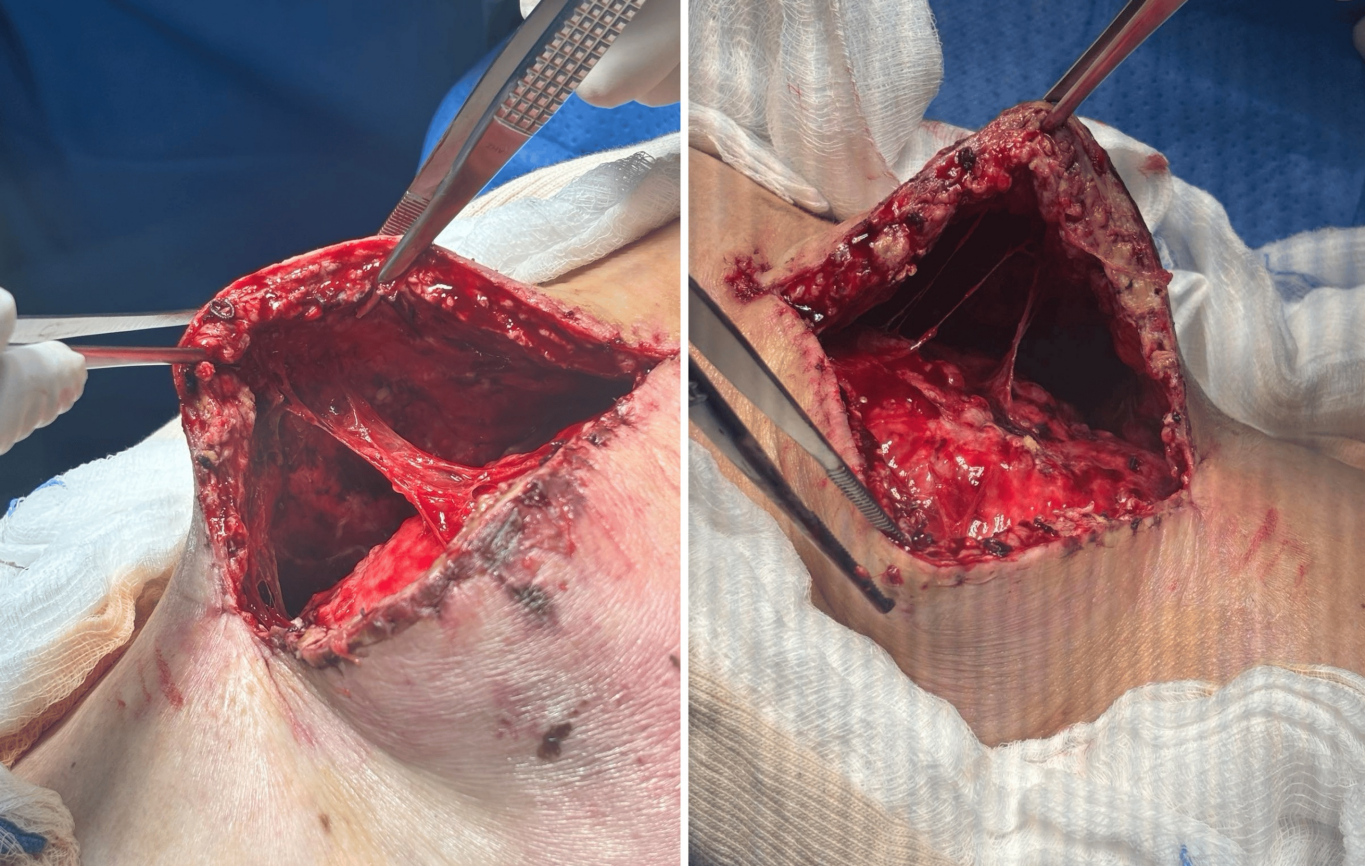
Intraoperative photographs of the closed degloving injury before debridement

Preoperative antibiotics were withheld. The patient was placed supine, and a midline incision, the same as for the primary TKA, was made. A large serosanguinous collection (~500 mL) was evacuated. The subcutaneous tissues were detached from the underlying fascia, and septations were also present (Figure 3). A closed degloving (MLL) injury was diagnosed intraoperatively. 

**Figure 4:**
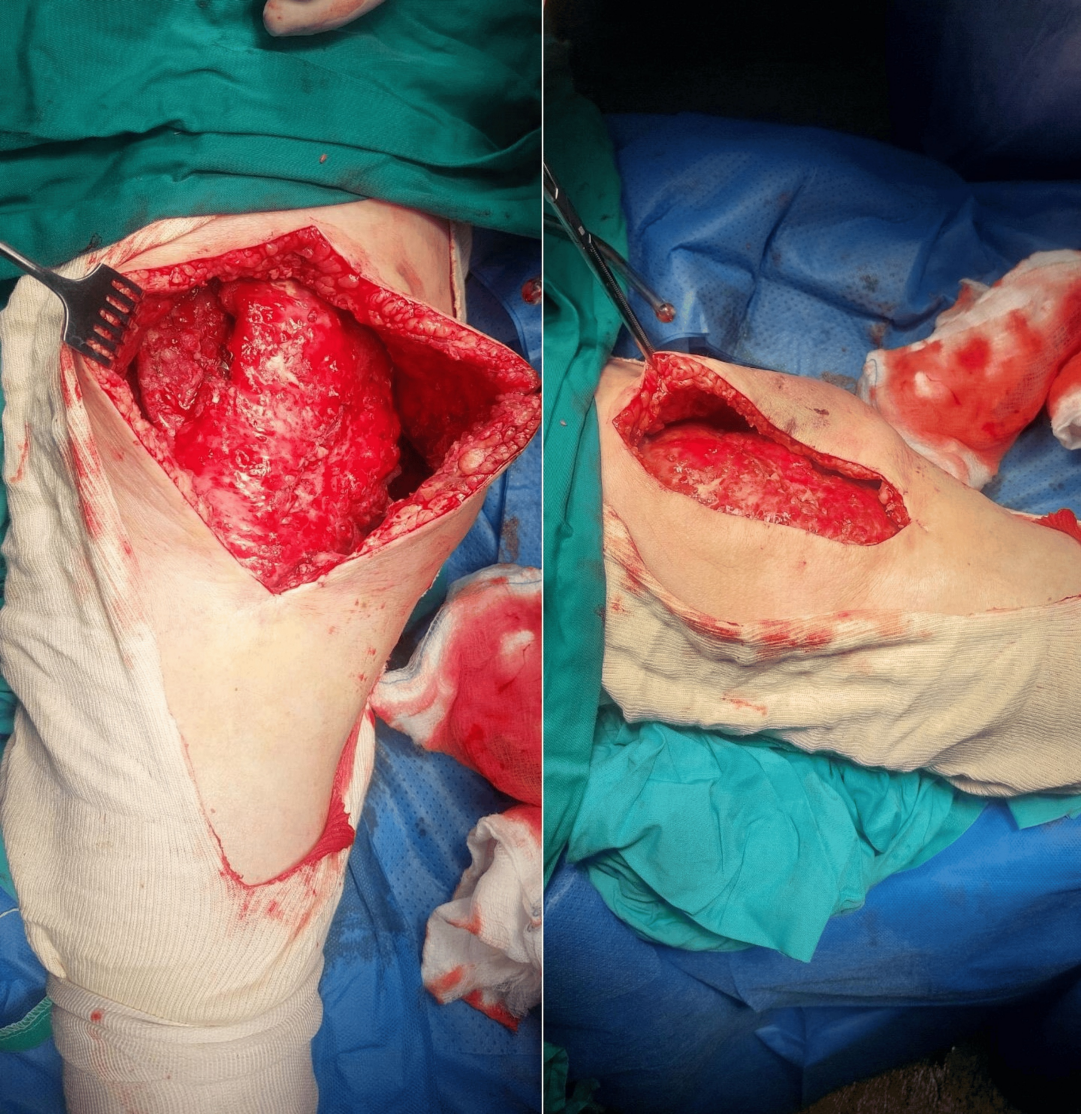
Intraoperative photographs after debridement of the subcutaneous layer and fascia to healthy tissue

**Figure 5:**
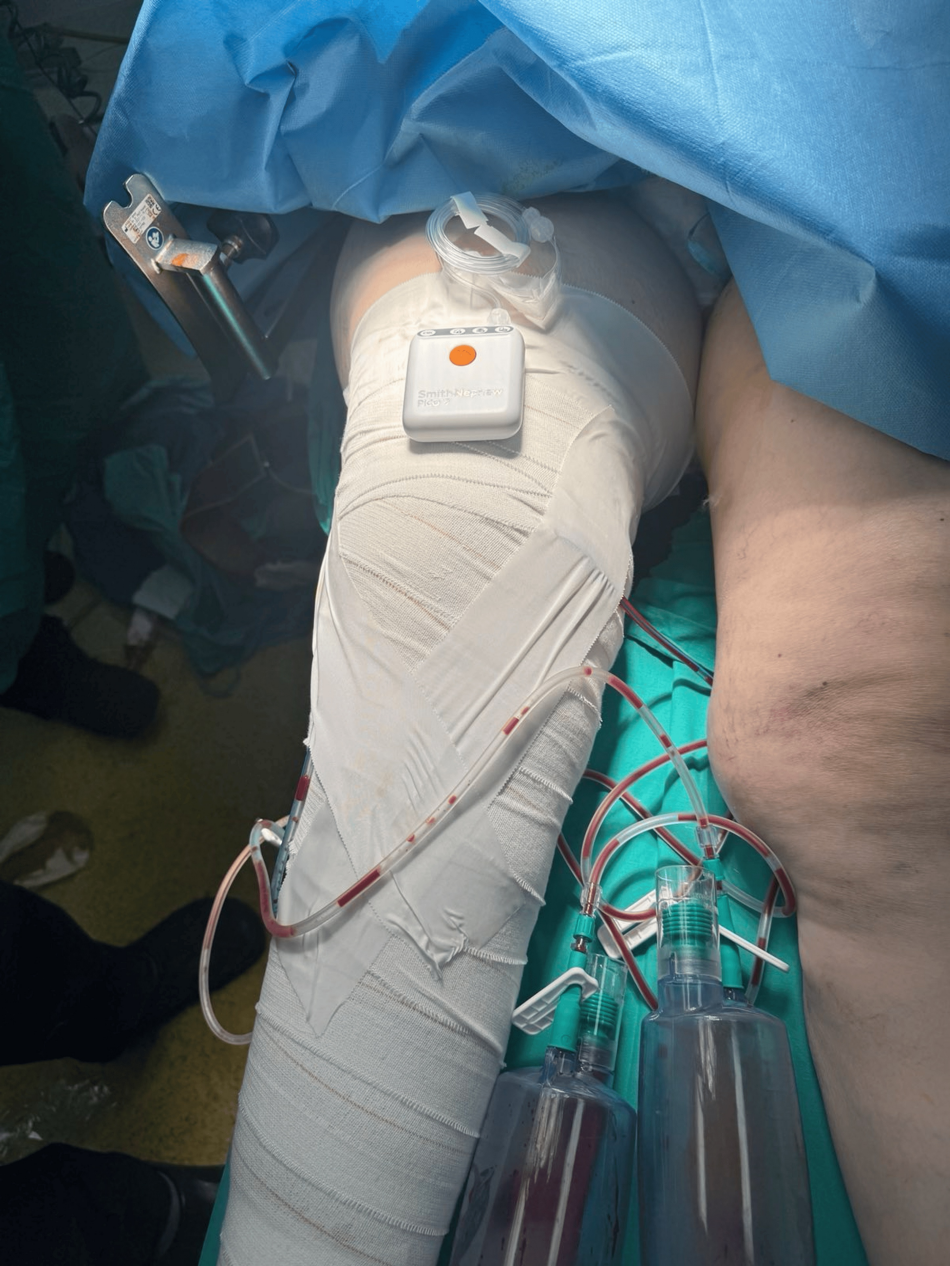
Postoperative picture showing the compression with adhesive bandage, drains and, incisional VAC (vacuum-assisted closure)

The cavity was scraped with a curette, and thorough lavage was performed. Tissue and fluid samples (six in total) were obtained for culture. After encountering healthy-looking, bleeding tissues (Figure 4), heavy braided Vicryl sutures were placed, transfixing the subcutaneous tissue to the fascia at intervals of 1-2 cm. Since this had been done during the previous I&D and because of the intraoperative diagnosis of MLL, the joint was not opened. Two drains were placed under negative pressure. The skin was closed with nylon sutures and an incisional vacuum-assisted closure (VAC). A high-compression adhesive bandage (Figure 5) was applied from the foot to the thigh. Empirical intravenous antibiotics (daptomycin 10 mg/kg once daily and cefepime 2 g three times daily) were then administered as per institutional protocol, pending the results of the tissue cultures.

**Figure 6:**
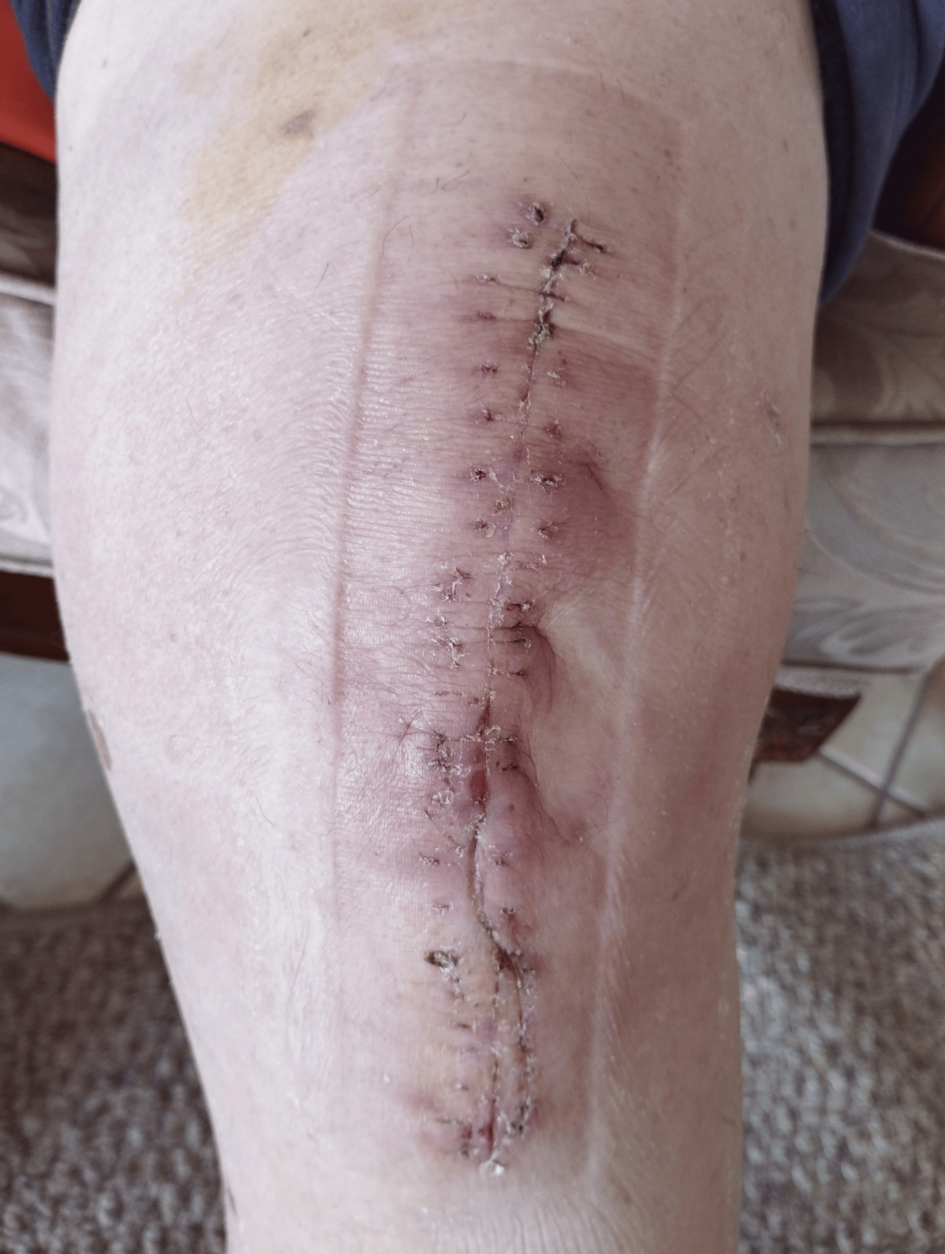
Photograph of the knee following suture removal

The postoperative course was uneventful. The dressings were changed on the third postoperative day, and one drain was removed. A high-compression bandage was then reapplied. The knee aspirate culture was negative. The incisional VAC and the remaining drain were removed on the seventh postoperative day. Tissue cultures were negative, and antibiotics were discontinued on the seventh postoperative day. The patient was then discharged. Sutures were removed three weeks postoperatively (Figure 6). The patient was instructed to use antithrombotic stockings.

At the three-month postoperative evaluation, the patient was walking without assistive devices, remained pain-free, and showed no evidence of recurrence.

## Discussion

MLLs were originally described by the French physician Morel-Lavallée in the 19th century. They involve traumatic disassociation of the muscle fascia from the comparatively more mobile superficial subdermal tissues and the creation of a dead space when tangential forces are applied [6]. They can also be referred to in the literature as "post-traumatic seroma" [7]. The injured perforating blood and lymphatic vessels lead to blood and lymph fluid accumulation, accompanied by necrotic fat and tissue debris [8]. The lesion eventually becomes enclosed in a fibrous capsule [9].

Diagnosis of an MLL remains clinical, although ultrasound [10], CT [11], or MRI [12] can be used reliably. A fluctuant mass with mobile skin is usually visible, often accompanied by adjacent contusions or abrasions [7]. Loss of sensation in the affected area may also occur [13]. In MLLs of the knee, 41% of patients had knee flexion deficits [14]. This was also consistent in our patient. About one-third of cases may be missed at presentation [15]. It may be difficult to differentiate from a prepatellar bursitis both clinically and radiologically [12]. Fluid collections in knee MLLs tend to extend beyond the margins of the prepatellar bursa [16]. An MRI classification of MLLs was developed, recognizing six subtypes, although its clinical significance remains unclear [17]. In an MRI study of 24 patients with knee MLL, the authors found that a predilection for the medial side of the knee may exist [16].

Judet and Letournel reported an incidence of 8.3% among 275 patients with injuries about the trochanter; smaller fluid collections may have been missed [13]. Trochanteric regions comprise 50% of MLL cases, while injuries at the scalp, thoracolumbar region, flank, and upper limb have also been reported [15,18,19], usually as a result of high-energy trauma [20]. The knee accounts for 15.6% of reported lesions [19]. For knee MLLs in athletes, a shearing blow to the knee appears to be the most common mechanism of injury [14]. Many reports have described MLLs after minor trauma or even without a recalled injury [21,22]. A case of a long-distance runner who developed an MLL after a marathon has also been reported [23]. This insidious onset and delayed presentation may mislead the clinician toward other pathologies such as abscess, hematoma [24], muscle contusion [25], or mass [26-28]. In our case, it resembled a prosthetic joint infection with pain, stiffness, and swelling. Thus, we did not consider an advanced imaging modality such as MRI preoperatively. In the case of aggressive joint mobilization, we could assume that the pathogenesis fits the repetitive microtrauma pattern.

Treatment of these injuries is critical. Specifically, Hak et al., in their review of 24 patients, found that 46% of tissue cultures were positive at the time of debridement [29]. Judet and Letournel recognized this potential and suggested debridement upon diagnosis or at the time of internal fixation [13]. This may pose a therapeutic challenge if there are underlying fractures or, as in our case, a prosthetic joint or other orthopedic hardware. Separation of the perforating vessels may also cause necrosis of the overlying skin [15], while continued fluid accumulation may further compromise vascular supply [29]. Aspiration of more than 50 mL was associated with an 83% recurrence rate [7], necessitating operative treatment for larger or recurrent effusions. Furthermore, MLLs have been reported to potentiate septic shock or cause significant bleeding requiring reoperation [30,31].

MLLs of the knee are less prevalent. Conservative treatment [14,23,24,32-35] with aspiration and/or compressive bandages seems sufficient for smaller lesions [16,33-37]. Compression is an important aspect of conservative therapy [38]. Tejwani et al., in the largest MLL knee series to date, reviewed 27 lesions in professional football players [14]. Fifty-two percent of knees were treated with conservative measures, mainly compression wrapping, cryotherapy, and motion exercises, with resolution of the lesion in about 10 days [14]. These patients likely had smaller effusions. The remaining knees were treated with aspirations, about half of which required additional aspirations (22%). Three cases eventually required sclerodesis with doxycycline, with no reported complications [14]. Even in larger MLLs, no operative treatment was undertaken, which may partially be attributed to the elite athletes’ desire to return to play without a prolonged convalescence period [14].

Regarding operative treatment, open surgery has traditionally been considered the gold standard. Open debridement and closure of dead space with sutures or negative pressure wound therapy (NPWT) have been successful [39,40]. Less invasive approaches were developed later, preserving the blood supply of the skin in the degloved area [41]. These include limited incision, debridement, and compression or drain placement [15,41]. Percutaneous quilting sutures can also be utilized with limited incision debridement [18]. More recently, arthroscopic-assisted drainage has been introduced [42,43], with the additional advantage of suture placement under direct vision. Our concern for infection necessitated an extensile incision for the washout. The large volume of evacuated hematoma justified this approach.

Evidence for the surgical treatment of knee MLLs remains limited. Emphasis is placed on thorough debridement and elimination of the dead space. Heifner et al. treated a recurrent MLL with open debridement, doxycycline, and tight closure with nylon sutures [21]. Van Gennip et al. described successful treatment in three patients: one with corticosteroid injection and rest, one considered to have an overuse injury requiring activity modification, and one who underwent endoscopic debridement [23]. Koc et al. treated a similar patient with a refractory suprapatellar MLL via endoscopic debridement with additional fibrin glue sclerotherapy [44]. Endoscopic debridement and sclerotherapy with doxycycline were also used by Kim for the treatment of a chronic MLL lesion in a 14-year-old patient [45]. Endoscopy reduced the morbidity of open exposure and prevented cosmetic complaints in the young patient. Weiss et al. debrided a large 26 × 13.8 × 6.2 cm MLL that had been aspirated and drained of about 1,900 mL [25]. The patient underwent two I&Ds with delayed primary closure six weeks after presentation [25]. Finally, similar to our case, Vanhegan et al. used internal quilting sutures, transfixing the fascia to the subcutaneous tissue in an open procedure for an MLL that had failed conservative treatment [19].

Other authors have used sclerosing agents such as ethanol [46], talc [47], or synthetic glue [48] in peripelvic MLLs, either with surgery or aspiration. Bansal et al. treated 16 patients with chronic MLLs using aspiration and doxycycline sclerotherapy [49]. One patient required repeat aspiration at eight weeks because of noncompliance with compression instructions [49]. This is a clear drawback, as prolonged compression is necessary until fibrosis is achieved. Furthermore, possible adherence of the skin and subcutaneous tissue to the fascia and muscle may cause functional complaints [49]. Fibrosis could compromise the function of the joint, which already presented with stiffness and decreased range of motion in our patient. We used compression bandaging, although not as a mainstay of treatment.

Postoperative seromas share similar characteristics with MLLs. Occurring in a potential space, they may serve as a site of infection [50], and there is no consensus on their treatment [51]. NPWT has been used to prevent seromas after total hip arthroplasty [52], although its effect on seroma occurrence remains ambiguous [53]. Postoperative seromas are frequent complications in abdominal and breast surgery [54,55]. Quilting sutures have been successfully employed by breast surgeons to reduce their occurrence post-mastectomy [56]. It is also difficult to predict which suprafascial hematomas will progress to MLLs, at least in the traumatic context [7]. In orthopedic surgery, most procedures do not involve soft tissue resection or the creation of significant dead space. While meticulous surgical technique and hemostasis are imperative in TKA to reduce complications [4], it is impossible to ascertain this regarding previous procedures. Surgeon experience does not appear to correlate with seroma occurrence [54]. Therefore, degloving could have resulted from either procedure and remained undiagnosed. Moreover, in the presence of a moderate postoperative suprafascial effusion, aggressive muscle stretching, as described, could have potentiated the shearing forces that led to the occurrence of an MLL. In the setting of a multiply operated infected TKA, Bettiol et al. used a porcine urinary bladder matrix to close the potential space after excising an encapsulated seroma [50]. A large wound bed with a soft tissue defect may have necessitated the use of the biologic substitute, which was not the case for our patient; the original TKA was also not infected.

## Conclusions

To our knowledge, this is the first reported case of a closed degloving injury following a TKA. While postoperative fluid collections are not uncommon after TKA, there have been no reports of traumatic separation of the tissue layers to this extent. The surgeon must maintain a high level of suspicion and include MLLs in the differential diagnosis of persistent or atypical postoperative knee swelling, especially after aggressive rehabilitation maneuvers, as MLLs can lead to pain, cosmetic deformity, and infection, causing considerable morbidity and the need for further operative intervention.
